# Effect of Primary vs Secondary Amines on the Reactivity and Dynamics of CO_2_ in Polyallylamine Sorbents under Humid Conditions

**DOI:** 10.1021/acsapm.6c00437

**Published:** 2026-06-22

**Authors:** Avery E. Baumann, Alice Klapproth, Richard A. Mole, Craig M. Brown, Christopher M. Stafford, Christopher L. Soles

**Affiliations:** † Materials Science and Engineering Division, 10833 National Institute of Standards and Technology, Gaithersburg, Maryland 20899, United States; ‡ 5419 Australian Nuclear Science and Technology Organisation, Lucas Heights 2234, New South Wales, Australia; § Center for Neutron Research, National Institute of Standards and Technology, Gaithersburg, Maryland 20899, United States

**Keywords:** carbon capture, sorption, humidity, jump diffusion, dynamics, quasielastic neutron scattering, infrared spectroscopy

## Abstract

Selective facilitated transport membranes are viable technologies for separating dilute CO_2_ from mixed-gas streams. In this study, we measure the reactivity and transport behavior of two polyallylamine amine polymers that bind CO_2_ in humid environments. We quantify sorption of CO_2_ and water components using a tandem quartz crystal microbalance mass sensor coupled with infrared spectroscopy for chemical identification of the sorbed gases and their reaction products. The two high-molecular-mass (glassy) polyallylamine materials, one containing just primary amines and the other containing isopropyl-functionalized secondary amines, react with humid CO_2_ to form tethered carbamate/carbamic acid groups or bicarbonate ions, respectively. Because our technique allows for the discernment of specific water and CO_2_ uptake, we also identify that the different reactivities influence the sorbed water content in the polymer films. We further quantify the local or segmental mobility of the dosed polymers with quasielastic neutron scattering measurements on the few nanoseconds to tens of picoseconds time scales, revealing that a classic jump diffusion model describes the dynamics of the polymers and their polymer–sorbate complexes. The resulting jump lengths and residence time between jumps differ based on the polymer chemistry and dosing conditions, with the hindered secondary amine systems generally presenting longer residence times and slightly longer jump distances. This combination study featuring both quasielastic neutron scattering and tandem gravimetric and chemical uptake measurements adds to the broader understanding of amine polymer dynamics and reactivity in CO_2_ capture applications.

## Graphical abstract

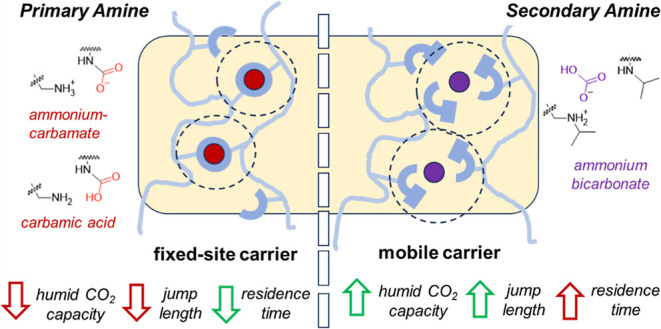

## Introduction

Different aminopolymers relevant for CO_2_ sequestration applications have been investigated in a myriad of device structures, including as sorbents within porous solid supports and within thin-film composite membranes.^1–5^ The nature of the aminopolymer utilized in each situation varies with the particular application and feed stream. For example, low to medium molecular mass hyperbranched polyethylenimine (PEI) is widely used as a sorbent material as it has low viscosity to facilitate CO_2_ diffusion and reacts to capture CO_2_ as carbamate. However, the soft and fluid nature of PEI necessitates it be housed in a solid support, which can impede sorption from both a physical mass transport perspective as well as by inducing a chemical “dead layer” that does not participate in CO_2_ capture.^6–10^ On the other hand, higher molecular mass polyvinyl- and polyallylamine polymers have been favored for dense polymer membrane technologies because they have high amine density, can be easily processed in thin-film composites as blends with structural polymer additives, and are often more stable than PEI.^5^

Facilitated transport membranes (FTM) have been investigated as a viable technology to separate CO_2_ from other gases in a mixed feed stream. As membranes need to mechanically separate the feed from the permeate side of the gas flow, they tend to be made of a higher molecular mass polymer that provides more structural rigidity. FTMs selective to CO_2_ operate using a mobile or fixed carrier to transport the molecule of interest across the membrane. Molecules with high affinity for CO_2_, such as amine or hydroxide groups, can be incorporated into the membrane as fixed-site carriers or mobile carriers depending on whether they are covalently bonded to the polymer backbone or not. These FTM membranes are ideal for CO_2_ separation from low concentration streams, such as in flue gas or direct air capture, as the carriers react predominantly with CO_2_ and require little or no energy to separate CO_2_ passively.^11–13^ FTMs may simultaneously allow for solution-diffusion of CO_2_ (and other gases), such that the total permeation of CO_2_ is a combination of Fickian diffusion and facilitated transport.^11^

Ho and co-workers published an inspiring collection of reports tuning the transport properties of poly­(vinylamine) and poly­(allylamine) FTMs using a series of alkyl functional groups. They found that the CO_2_ transport mechanism and FTM performance are highly dependent on the amine chemistry.^14,15^ The mechanism of transport affects the overall performance of the membrane, as demonstrated in one example where membranes containing unhindered (primary) amines in a polyallylamine (PAA) membrane are ∼4× less selective for CO_2_/N_2_ and ∼6× less permeable than analogous poly­(*N*-isopropyl allylamine) (PAAR) membranes containing hindered (secondary) amines.^14^ The authors attribute the difference in membrane performance to differences in transport mechanisms, hypothesizing that the hindered amine converts CO_2_ to a mobile bicarbonate ion, which moves through the membrane via a bound carrier (hydroxide), while the unhindered amine relies on facilitated CO_2_ “hopping” from one amine bonding site to another via carbamate ion/carbamic acid formation. Their reactivity and proposed transport pathways for PAA-HC and PAAR-HC (where “-HC” denotes the sample is dosed with water and CO_2_) are illustrated in Scheme 1. We draw the PAAR-HC sample with some unhindered amines that are more likely to form carbamate/carbamic acid sorption products, as supported by our infrared spectroscopy measurements.

**Scheme 1:**
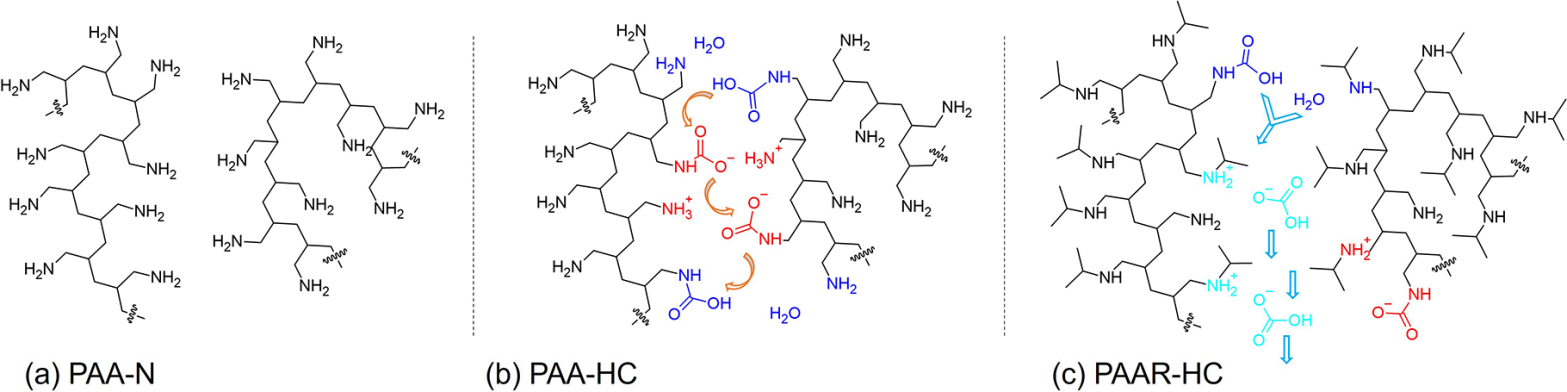
Structure Representations of (a) PAA-N (PAA Dosed with N_2_ Only), (b) PAA-HC (PAA Dosed with H_2_O and CO_2_), and (c) PAAR-HC (PAAR Dosed with H_2_O and CO_2_)­^a^

a The PAA-HC sample forms carbamate ions charge-balanced by ammonium neighbors (red) or carbamic acid (blue) via proton shuttling through sorbed water that hop from amine to amine during transport. The hindered PAAR-HC sample predominantly forms a bicarbonate species (aqua) that is not bound to the polymer network, but unhindered amines can still form carbamate/carbamic acid moieties (red/blue, respectively) as well. The scheme depicts only the central corridor of amines reacting, but all accessible amines are suitable for interaction with water and/or CO_2_.

Motivated by the proposed differences in the transport mechanisms and apparent membrane CO_2_ separation performance, here we present a model study of the dynamics in two systems studied by Ho and Zhao^14^ using quasielastic neutron scattering (QENS). We present a model study of the transport mechanisms of the same PAA and PAAR materials presented in their previous manuscript; this is a mechanistic study to increase the understanding of these different materials and we do not attempt to further the deployment of these materials as FTMs. QENS is a powerful technique for this mechanistic study because it is sensitive to the local (sub 2 nm) length scales and is exquisitely sensitive to the dynamics of hydrogen-containing moieties in the membrane owing to hydrogen’s large incoherent neutron scattering cross section with respect to other elements. QENS has proven to be insightful for understanding the dynamical differences in PEI sorbents both with^7,9,16^ and without^17^ a solid support structure. In our previous work, we used QENS to assess the dynamics of PEI as a function of dosing with water and/or CO_2_. We found the center of mass diffusion of the polymer-sorbate system to move in a Fickian manner, with transport enhanced by water (both ^1^H_2_O and ^2^H_2_O) and CO_2_ on the local length scale.^17^ When codosed with water, CO_2_ slows down the dynamics relative to the water-only case, due to the formation of carbamate ions that form transient cross-links in the PEI. These findings serve as a starting point for the work presented here, where we examine the effect of dosing water and CO_2_ on two different aminopolymer systems with different reactive sorption behavior.

To provide chemical insight into sorption in these polyallylamines, we include uptake measurements using polarization modulation infrared reflection absorption spectroscopy (PM-IRRAS) in tandem with quartz crystal microbalance (QCM) gravimetric measurements^17–19^ that quantify sorption of both CO_2_ and water individually. In agreement with the mechanism proposed by Ho and Zhao, we identify the formation of bicarbonate and carbamate species in the hindered PAAR sample when dosed with humid CO_2_ whereas the unhindered PAA sample only forms carbamate moieties in analogous conditions. Taken together, the QENS and QCM/PM-IRRAS results characterize the effect of dosing water and CO_2_ on two different polyallylamine systems with different reactive sorption behaviors. Furthermore, the resulting physicochemical observations on glassy PAA and PAAR systems vary significantly from previous hyperbranched PEI studies, a rubbery polymer, and inform the structure–property relationship landscape for aminopolymer CO_2_ sorbent/membrane materials.

## Experimental Section

Certain commercial equipment, instruments, materials, or software are identified in this paper to foster an understanding. Such identification does not imply recommendation or endorsement by the National Institute of Standards and Technology, nor does it imply that the materials or equipment identified are necessarily the best available for their purpose.

### Materials

Poly­(allylamine hydrochloride) (PAA-HCl) was purchased from Alfa Aesar (120,000 g mol^–1^ to 200,000 g mol^–1^) and used as the starting material for both samples studied. PAA and PAAR were prepared following a previously published protocol.^14^ These are relatively high-molecular-mass polymers that would be relevant for FTMs. By contrast, our previous study on PEI focused on a lower-molecular-mass analog (800 g mol^–1^) that would be relevant to porous supported sorbents.^17^ To prepare the free base version of the amine, PAA, 1 equiv per amine of KOH (0.3 g, Talyor Chemical, 5.3 mmol) was added to 0.5 g of PAA-HCl (Alfa Aesar, 5.3 mmol, amine basis) along with 100 mL of methanol and stirred at room temperature for 48 h. In this process, the KCl generated precipitates from the methanolic solution and can be removed via filtration. The *n*-isopropyl group is incorporated to form poly­(*N*-isoproply allylamine) (PAAR) by adding 0.5 mL of 2-bromopropane (Alfa Aesar, 5.3 mmol) and 0.3 g of KOH (5.3 mmol) to the recovered PAA solution heated to 55 °C. The mixture was maintained at 55 °C for 48 h while the KBr is precipitated and removed via filtration. The remaining solution contains the reacted PAAR. For QENS measurements, a deuterated version of the isopropyl group was prepared to minimize the trivial, fixed center of mass methyl rotor motions (which should not facilitate transport) contributions to the scattering data. The synthesis procedure was identical except that deuterated 2-bromopropane (d7, 98%, Cambridge Isotope Laboratories) was used.

QENS samples were prepared by casting the PAA and PAAR into aluminum foil packets, dried passively at room temperature until the methanol had evaporated, and then further dried at 100 °C under vacuum (roughly 15 kPa) for 1 h. The packets were removed from the vacuum oven and immediately placed into mylar bags that were quickly sealed with a heat-sealing press. Inlets on the bags allowed us to dose the content of the bags with desired compositions (22% CO_2_ in N_2_ balance, 57% ± 5% relative humidity (RH) achieved via a ^1^H_2_O or ^2^H_2_O (D_2_O) bubbler). CO_2_- and H_2_O-dosed samples are referred to with the “HC” label, while CO_2_- and D_2_O-dosed samples are referred to with the “DC” label. The undosed sample packed under N_2_ is labeled “N”. All samples were sealed individually under positive pressure for transport.

### QCM/PM-IRRAS

Infrared spectra were collected using a Nicolet iS50 FTIR spectrometer (Thermo Fisher Scientific) with PM-IRRAS accessories and a liquid nitrogen-cooled mercury–cadmium-telluride detector. Samples for analysis were housed within a QCM cell (custom designed, AWSensors) discussed previously.^17–19^ The atmosphere in the cell was controlled using a combination of mass flow controllers (Alicat Scientific) and a water bubbler to tune the RH. The photoelastic modulator was centered at 2000 cm^–1^, and spectra were collected in a window of 1000 cm^–1^ to 4000 cm^–1^. Kinetic uptake spectra were collected using the series mode in the instrument software (OMNIC, Thermo Fisher Scientific) with a repetition time of 60 s. Each spectrum is comprised of 60 individual scans, averaged over a 60 s repeat acquisition time. Before dosing with sorbate gases (CO_2_, H_2_O), the sample was first heated under flowing N_2_ to 100 °C and then passively cooled to the appropriate temperature under flowing N_2_. The molar concentration of CO_2_ is 22% in N_2_ balance where applicable.

### QENS

For neutron experiments, the dosed polymer samples in their foil packets were quickly removed from the sealed Mylar bags, loaded, and hermetically sealed into concentric cylindrical QENS sample cans with an annular space of 0.1 mm. The PAA-N and PAAR-N (packed under dry N_2_) samples were loaded into the can under a N_2_ atmosphere to eliminate the introduction of H_2_O or CO_2_ from ambient air; we assume that this transfer procedure did not affect the dosing conditions of the sample. Once mounted in the QENS spectrometers, the temperature of the sealed sample cells was controlled during the measurement with a closed-cycle cryostat with a helium circulation loop controlled by a digital needle valve.

Neutron scattering on the Emu^20^ spectrometer was collected at a wavelength of 6.28 Å. Elastic scans were obtained by slowly ramping the temperature between 50 and 325 K and recording the elastic scattering intensities as a function of momentum transfer, *Q*. QENS measurements were obtained at 300, 325, and 340 K. For the quasi-elastic measurements, the speed of the Doppler drive that determines the energy range of the spectrometer was set to 4.7 m s^–1^ (9.962 Hz). This corresponded to a dynamic energy range of ±31 μeV over a *Q* range of 0.3 Å^–1^ to 1.8 Å^–1^. The data were reduced and binned in Mantid^21^ and subsequently analyzed using the DAVE^22^ software. QENS data were fit across the entire ±31 μeV energy range from *Q* = 0.5 Å^–1^ to 1.7 Å^–1^ in 0.2 Å^–1^ increments. The resolution function was collected at 6 K where polymer and small-molecule dynamics are assumed to be frozen. All data were corrected for background scattering and detector efficiency by subtracting data from an empty cell and using vanadium for normalization runs, respectively.

The same sample cans that were prepared for Emu measurements were also measured on the Pelican^23^ spectrometer using a wavelength of 5.98 Å and an instrument resolution of ≈65 μeV at temperatures of 300, 325, and 340 K and collected over a range of momentum transfers from *Q* = 0.25 Å^–1^ to *Q* = 1.85 Å^–1^ in 0.2 Å^–1^ increments. The data were reduced and binned in Mantid^21^ and analyzed using the DAVE^22^ software. The region with sufficient data for fit includes *Q* = 0.45 Å^–1^ to *Q* = 1.65 Å^–1^ for all samples; the spectra were cropped to include only data from −2 to 1.5 meV.

## Results & Discussion

### Chemical Uptake and Speciation

The original report^14^ by Ho and Zhao provides infrared spectra as chemical characterization of the PAA and PAAR materials. In that prior study, the N–H stretch is utilized as the diagnostic signal to ascertain the degree of alkyl functionalization of the primary amine. PAA is shown to have peaks at 3360 cm^–1^ and 3289 cm^–1^, while PAAR only had one band at 3312 cm^–1^. This agrees with the N–H region of our PAA (3350 cm^–1^ and 3265 cm^–1^) and PAAR (3320 cm^–1^) spectra shown in Figure 1a,b. Further thermogravimetric analysis (TGA) experiments were conducted to characterize the PAA and PAAR samples Figure S1. The TGA experiments were conducted in a N_2_ atmosphere up to 600 °C and then switched to air and heated to 1000 °C, after which there was <3% residual mass in the pan. There are small mass loss events below 400 °C in both samples, accounting for the loss of ammonia and hydrazine as reported previously.^24^ The bulk of the mass loss occurs upon the switch from N_2_ to air, whereby the remaining carbon is oxidized to CO_2_. PAAR contains more carbon due to installation of the isopropyl group and displays greater mass loss in the high-temperature region than PAA with approximate values of 68% and 46% of the initial dry mass, respectively. The degradation behavior below 400 °C is slightly different and reflects the change in chemistry in PAA vs PAAR.

**Figure 1:**
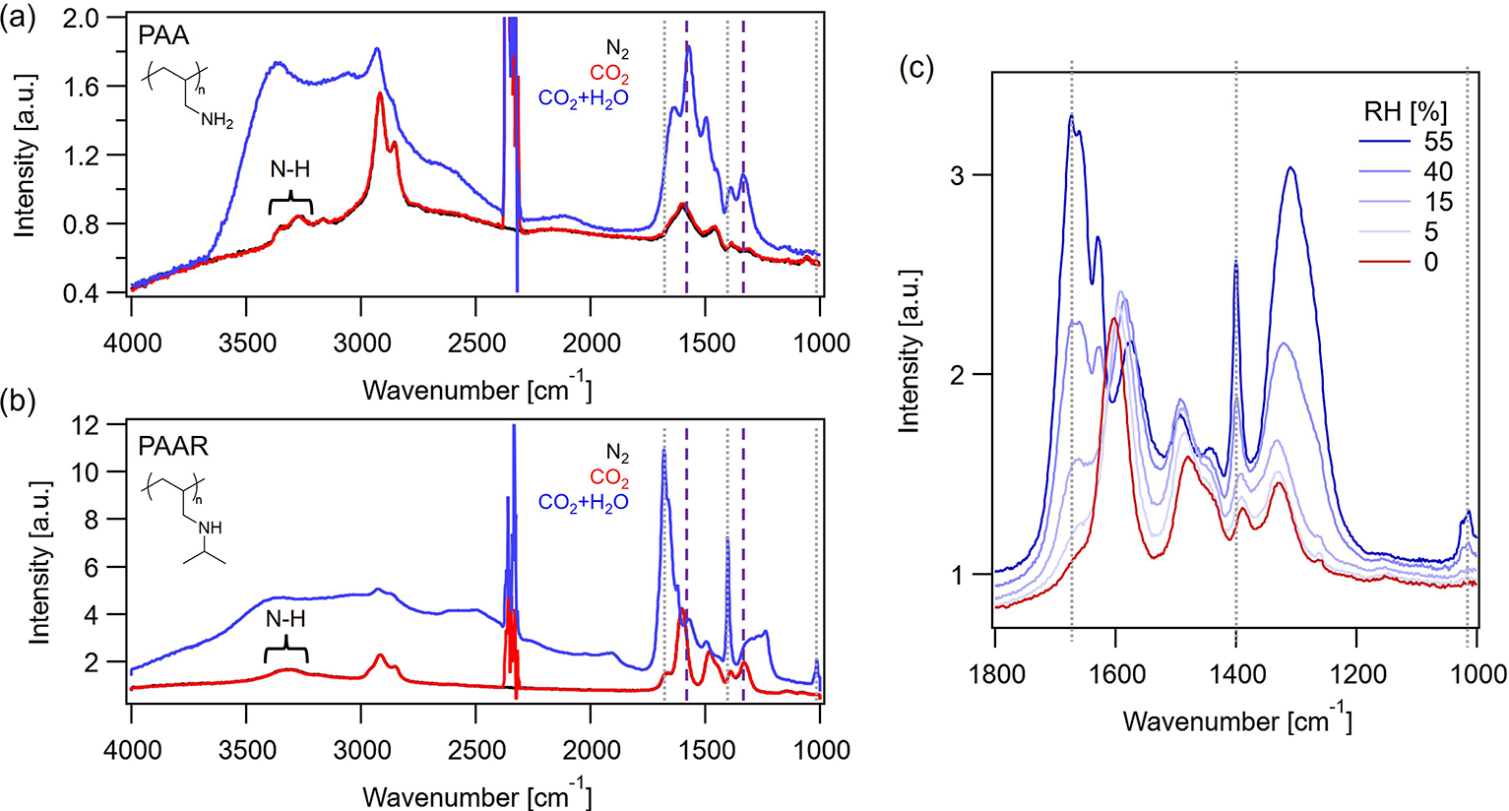
PM-IRRAS data showing (a) PAA and (b) PAAR under N_2_ (black), CO_2_ (red), and mixed H_2_O + CO_2_ (blue) environments. Lines highlight peaks attributed to bicarbonate (gray dotted line) and carbamate (purple dashed line) moieties. (c) PAAR dosed with 22% CO_2_ at the relative humidities indicated in the legend. Bicarbonate formation occurs in this series at 40% RH.

We examine the reactivity of the prepared films of PAA and PAAR to CO_2_ in both dry and humid conditions using PM-IRRAS, as shown in Figure 1a,b. Neither PAA nor PAAR shows chemical reactivity toward dry CO_2_ as the spectra remain unchanged in the carbonyl region, presumably because mobility in the dry state is limited such that appreciable CO_2_ does not penetrate the films. However, in mixed CO_2_–H_2_O atmospheres (22 molar % CO_2_ in N_2_ balance, 57% ± 5% RH achieved via H_2_O bubbler), carbamate and bicarbonate formation is evident in PAAR by associated peaks indicated by dashed and dotted lines in Figure 1a,b, respectively. These observations differ from our previous studies on PEI^17–19^ where carbamate could form without the presence of water and no bicarbonate signals were apparent with dry or wet CO_2_ exposure. Jones et al. previously reported a low-molecular-weight PAA (<1200 g mol^–1^) loaded into a silica foam sorbs CO_2_ from dry 10% CO_2_ balanced with Ar.^25^ However, the combination of low molecular mass (compared to >120,000 g mol^–1^ in this report) and the inclusion of a solid support structure could alter the polymer’s reactivity and dynamics such that the sorption behavior to dry CO_2_ differs in the two instances. Spectra from both PAA (Figure 1a) and PAAR (Figure 1b) samples dosed with humidified CO_2_ form carbamate, with peaks appearing at 1570 cm^–1^ and 1330 cm^–1^ (dashed lines). Additionally, formation of bicarbonate in the dosed PAAR sample is inferred from the evolution of peaks at 1675 cm^–1^, 1401 cm^–1^, and 1014 cm^–1^ (dotted lines). The formation of bicarbonate occurs through addition of water to CO_2_. In a study where the RH was controlled via H_2_O bubbler, we found that bicarbonate formation did not appear in the dosed PAAR spectra until RH was roughly 40% (Figure 1c), while carbamate was detected at lower RH values of 5% and 15%. We presume that even low RH dosing can enable CO_2_ capture in PAAR via a carbamate moiety, but that more local water near the amine sites (achieved at increasing RH) is necessary for bicarbonate formation. The differences in the reactivity of these two polyallylamines is the main thesis of the prior study^14^ wherein PAAR is believed to operate via transport of bicarbonate products and PAA relies on fixed-carrier carbamate hopping along the amine backbone as drawn in Scheme 1.

We examine the uptake of individual components using a custom-built QCM/PM-IRRAS chamber. The QCM quantifies the total mass of the absorbed gas while the PM-IRRAS enables speciation of this total mass into the individual components through spectroscopic signatures of sorbed molecules. In our previous QCM/PM-IRRAS studies on PEI,^17–19^ the dry CO_2_ uptake was used to define the relationship between the carbamate peak around 1310 cm^–1^ and the chemisorbed mass of CO_2_. Since no appreciable carbamate is formed under dry CO_2_ in these samples, we instead quantify the amount of water sorbed using the peak intensity at 3400 cm^–1^. This method can be less precise as the O–H peak at 3400 cm^–1^ is very broad and shifts slightly due to the local hydrogen bonding environment of the water, but still provides a handle to separate the water and CO_2_ uptakes for our purposes (see the Supporting Information for calculation). Furthermore, this method also has the advantage that it does not matter if the CO_2_ forms carbamate or bicarbonate ions; both are apparent in the spectra presented in Figure 1. We present the relationship between gravimetric water uptake and the 3400 cm^–1^ peak intensity in an experiment where films are exposed to humidified N_2_ in Figure S2. This calibration curve allows us to directly calculate a mass fraction of water (*M*_pH_2_O_) and indirectly estimate a mass fraction of CO_2_ (*M*_pCO_2__) via subtraction from the total mass (*M*_tot_).

Results from tandem QCM/PM-IRRAS experiments enable us to estimate the fraction of sorbed water and CO_2_ by each of the samples in experiments with different dosing protocols (Figure 2). We conducted two sets of experiments where the two different films were exposed to (i) first humidified N_2_ followed by humidified CO_2_ (Figure 2a) or (ii) first dry CO_2_ followed by humidified CO_2_ (RH = 60% ± 5%) (Figure 2b,c), where after each change in the dosing gas the system was allowed to equilibrate for 1 h.

**Figure 2:**
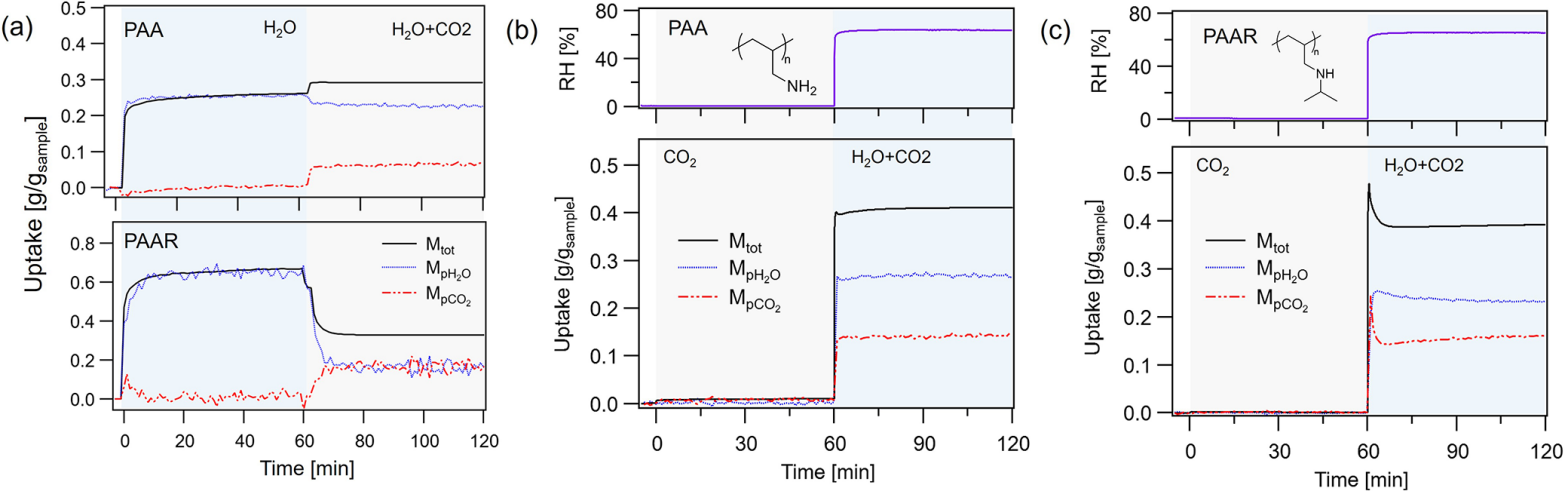
QCM/PM-IRRAS experiments allow for deconvolution of component uptake by the polymer films. The different traces in each panel correspond to component masses of water (*M*_pH_2_O_, blue, dot line) and CO_2_ (*M*_pCO_2__, red, dash-dot line) within the total mass (*M*_tot_, black, solid line). (a) PAA (top) and PAAR (bottom) samples are first exposed to humidified N_2_ and then humidified CO_2_ for 1 h each at 25 °C. (b) PAA and (c) PAAR dosed with dry CO_2_ for 1 h, then humidified CO_2_ for 1 h at 25 °C. The RH is plotted above in panels (b,c).

The estimated uptake values at the end of the 1 h of dosing stage under each specified condition are listed in Table 1. For PAA, the value of *M*_pH_2_O_ (in g/g_sample_) is nominally constant between 0.23 and 0.27 for all humidity experiments, regardless of whether there is CO_2_ present in the dosing gas or not. This suggests, to a first approximation, that there is no competition between the CO_2_ and water uptake. In contrast, for the PAAR films we see that *M*_pH_2_O_ decreases significantly from roughly 0.69 g/g_sample_ to 0.17 g/g_sample_ when CO_2_ is introduced to the prehumidified sample (Figure 2a). This is consistent with the hypothesis that CO_2_ is converted to a bicarbonate species in PAAR via a reaction that consumes water, thereby lowering the associated water O–H signal used to calculate *M*_pH_2_O_. We also note that when the CO_2_ is introduced into the humid environment, the PAAR film picks up about twice as much CO_2_ as the PAA film (0.16 g/g_sample_ and 0.07 g/g_sample_, respectively). The theoretical capture efficiency of the bicarbonate mechanism is exactly twice the bicarbonate, which could be part of the explanation for doubled CO_2_ uptake in PAAR vs PAA samples. Figure 1 shows that PAA is dominated by carbamate and PAAR is dominated by bicarbonate capture products, supporting this notion.

**Table 1:** Estimated Uptake Values for Mass of CO_2_ (*M*_pCO_2__) and H_2_O (*M*_pH_2_O_) Components for PAA and PAAR Samples Determined from Tandem QCM/PM-IRRAS Experiments^a^

	estimated uptake at 1 h (g/g_sample_)			
experiment	PAA *M*_pCO_2__	PAA *M*_pH_2_O_	PAAR *M*_pCO_2__	PAAR *M*_pH_2_O_
CO_2_	0	NA	0	NA
H_2_O	NA	0.26 ± 0.03	NA	0.69 ± 0.07
CO_2_ saturated to CO_2_–H_2_O	0.15 ± 0.02	0.27 ± 0.03	0.16 ± 0.02	0.23 ± 0.02
H_2_O saturated to CO_2_–H_2_O	0.07 ± 0.01	0.23 ± 0.02	0.16 ± 0.02	0.17 ± 0.02

a Values are normalized to the mass of the polymer sample.

The QCM can also be operated in “dissipation” mode (QCM-D) where a dissipation factor comes from the imaginary component of the complex electrical admittance of the resonator operating at approximately 5 MHz. These high-frequency dissipation measurements are defined in the Supporting Information and reported in Figure S3 under these same dosing conditions as the mass uptake curves in Figure 2. We observe a significant softening in PAAR films upon exposure to humidified N_2_, but surprisingly very little change in the PAA sample despite significant water uptake measured gravimetrically. This seems to suggest that the water cannot fully disrupt the existing network of intra- and intermolecular associations in PAA. When the CO_2_ is then introduced into the hydrated films, the carbamate formation in the PAA would induce cross-links that keep the film stiff and resist swelling. In contrast, in the PAAR sample we measure a precipitous decrease in the dissipation when the CO_2_ is introduced to the hydrated film. As the formation of bicarbonate species consumes water, we believe that the stiffening is attributed to decreased water that is available to plasticize the PAAR; the bicarbonate species do not appear to soften the film. Even though our experiments are conducted under flowing gas, more water does not partition into the PAAR film after CO_2_ is introduced. This suggests a change in *M*_pH_2_O_ saturation occurs upon reaction with CO_2_, potentially related to the stiffening of the film.

There is negligible sorption of dry CO_2_ by either PAA or PAAR polymers (Figure 2b,c). Sorption of CO_2_ only takes place when water vapor is introduced at 25 °C. The mass of water (*M*_pH_2_O_, blue) and CO_2_ (*M*_pCO_2__, red) is separated within the total mass (*M*_tot_, black) using the calibration curve and calculation described in the Supporting Information. The CO_2_ capacity of the PAA sample was estimated by subtracting *M*_pH_2_O_ from *M*_tot_ (the total mass measured by the QCM) to yield *M*_pCO_2__ of 0.15 g/g_sample_ or 0.07 g/g_sample_ depending on whether the film was first saturated with CO_2_ or water, respectively. The decreased CO_2_ uptake in the prehydrated PAA samples is interesting. We know that in PAA the carbamate is the predominant capture product formed via reaction at the amine sites. When the film is first saturated with water, some of these polar amine sites are less accessible to incoming CO_2_ and therefore the capture capacity is decreased, consistent with our previous study where, at 300 K, we observe roughly a 60% decrease in CO_2_ uptake if PEI is first exposed to humidified N_2_.^17^

The PAAR sample forms bicarbonate products in addition to carbamate based on the PM-IRRA spectra in Figure 1. In the QCM/PM-IRRAS profiles, there is a spike in total mass uptake upon introduction of CO_2_ to the sampling cell in the PAAR experiment that is also apparent in the *M*_pCO_2__ curve. The spike represents a real change in the total mass (*M*_tot_); however, it is unclear whether this mass is attributed to an increase in *M*_pH_2_O_ or *M*_pCO_2__, since these quantities are estimated from PM-IRRAS data collected over the course of 1 min (whereas *M*_tot_ is measured from QCM roughly every 0.8 s). An increase in the water signal does increase *M*_pH_2_O_, followed by a more gradual decrease with continued bicarbonate formation. A similar trend in water signal was observed in our studies on inorganic alkali hydroxides,^26^ where carbonates form in the presence of humid CO_2_. After 1 h, considerably less water remains in the PAAR film in the codosed CO_2_–H_2_O experiments (roughly 0.2 g/g_sample_) than the single-component experiment (0.69 g/g_sample_) in consequence. Since bicarbonate can be released from the amine site after formation, there should be less competition for amine sites than in the carbamate-dominated capture pathway. Without competition for amine sites, it is not surprising that the mass of CO_2_ captured after 1 h of exposure is the same regardless of the presaturation condition in PAAR, which is favorable for applications where water tolerance in the feed gas stream is desired. Armed with this understanding of composition and sorption differences in the dosed PAA and PAAR samples, we next focused on the dynamic behaviors of the systems.

### Quasielastic Neutron Scattering

QENS probes local molecular motions on the nano- to picosecond time scale. QENS is ideal to study polymer and sorbate dynamics owing to the large incoherent scattering cross section of ^1^H, which is prevalent in the backbone of both PAA and PAAR, and both polymer and small-molecule dynamics can be measured using QENS instruments. The time scales of translational center of mass motions of the macromolecules are best studied using backscattering spectrometers that have an energy resolution in the 1 μeV to 30 μeV range, corresponding to roughly a 10 ps to 1 ns time scale; these motions are slow enough to be dominated by diffusive processes. Time-of-flight spectrometers offer dynamic energy ranges from roughly 50 μeV to 1 meV and are better suited to quantify the much faster and local process occurring on the subpicosecond to 10 s of picosecond time scale. At the Australian Centre for Neutron Scattering (within Australia’s Nuclear Science and Technology Organisation, ANSTO), we measured the same series of dosed samples on both Emu^20^ (backscattering) and Pelican^23^ (time-of-flight) neutron spectrometers that encompass these time scales.

Elastic scans on the Emu spectrometer, sometimes referred to as “fixed-window” scans, provide information about the total proportion of dynamics with respect to the energy resolution of the spectrometer. In these elastic scans, the overall intensity of the elastic peak (*I*(*Q*,*T*)) is recorded as a function of momentum transfer, *Q*, and temperature, *T*. As the sample is heated, the intensity of the elastic scattering decreases due to harmonic-like vibration at low temperature *T* as well as the onset of anharmonic dynamic processes. In these elastic scans, the Doppler drive that helps discern the energy distribution of the scattered neutrons is turned off; the only indication of the time-scale of the dynamics is that processes are faster than the 1 μeV (4 ns) resolution of the instrument. These elastic scans are useful to determine at what onset *T* the dynamics become active. The intensity of the elastic scattering at each temperature, *I*(*Q*,*T*), is normalized to the scattering at a cold enough temperature to assume dynamics are sufficiently suppressed (here, 50 K) and converted to a mean squared atomic displacement at a given temperature, ⟨*u* ^2^(*T*)⟩, over the momentum transfer range *Q* (in Å^–1^) via the Debye–Waller approximation assuming a Gaussian distribution of displacements

1 $$-3\ln\left(\frac{I\left(Q,T\right)}{I_{50K}\left(Q,T_{50K}\right)}\right)\approx Q^{2}\left[\langle u^{2}\left(T\right)\rangle \right]$$

The change in elastic intensity corresponds to some relaxational motion entering the time window of the instrument, which on Emu is roughly 0.5 to 3.8 ns.^20^

Figure 3 shows the resulting ⟨*u* ^2^(*T*)⟩ curves for PAA and PAAR samples after dosing first with a stream of dry nitrogen (–N) followed by a combined stream of CO_2_ and H_2_O vapor (-HC) or CO_2_ and D_2_O (-DC). This protocol varies slightly from the QCM-PMIRRAS experiments where we varied the order of dosing with the different gases. As mentioned previously, the QENS signal is dominated by the hydrogen containing moieties in the sample. As CO_2_ does not have any protons, its dynamics are not readily seen in QENS; rather we see the effects that the CO_2_ has on the dynamics of the polymer. H_2_O adds protons to the system, so additional dynamic processes are introduced when dosing with H_2_O. However, we can in general separate the dynamics of the H_2_O from the polymer by using D_2_O. By comparing the dynamics of the dry polymer to the polymer dosed with D_2_O, we can estimate how hydration (assuming H_2_O and D_2_O are chemically equivalent) facilitates the dynamics of the polymer. The additional dynamics (relative to the D_2_O dosed sample) that arise in the presence of H_2_O represent the dynamics of the water molecules themselves and any mobile proton-containing capture products that are created. In the remainder of this manuscript the sample nomenclatures N, C, H, and D refer to dosing with dry N_2_, CO_2_, H_2_O, and D_2_O, respectively.

**Figure 3:**
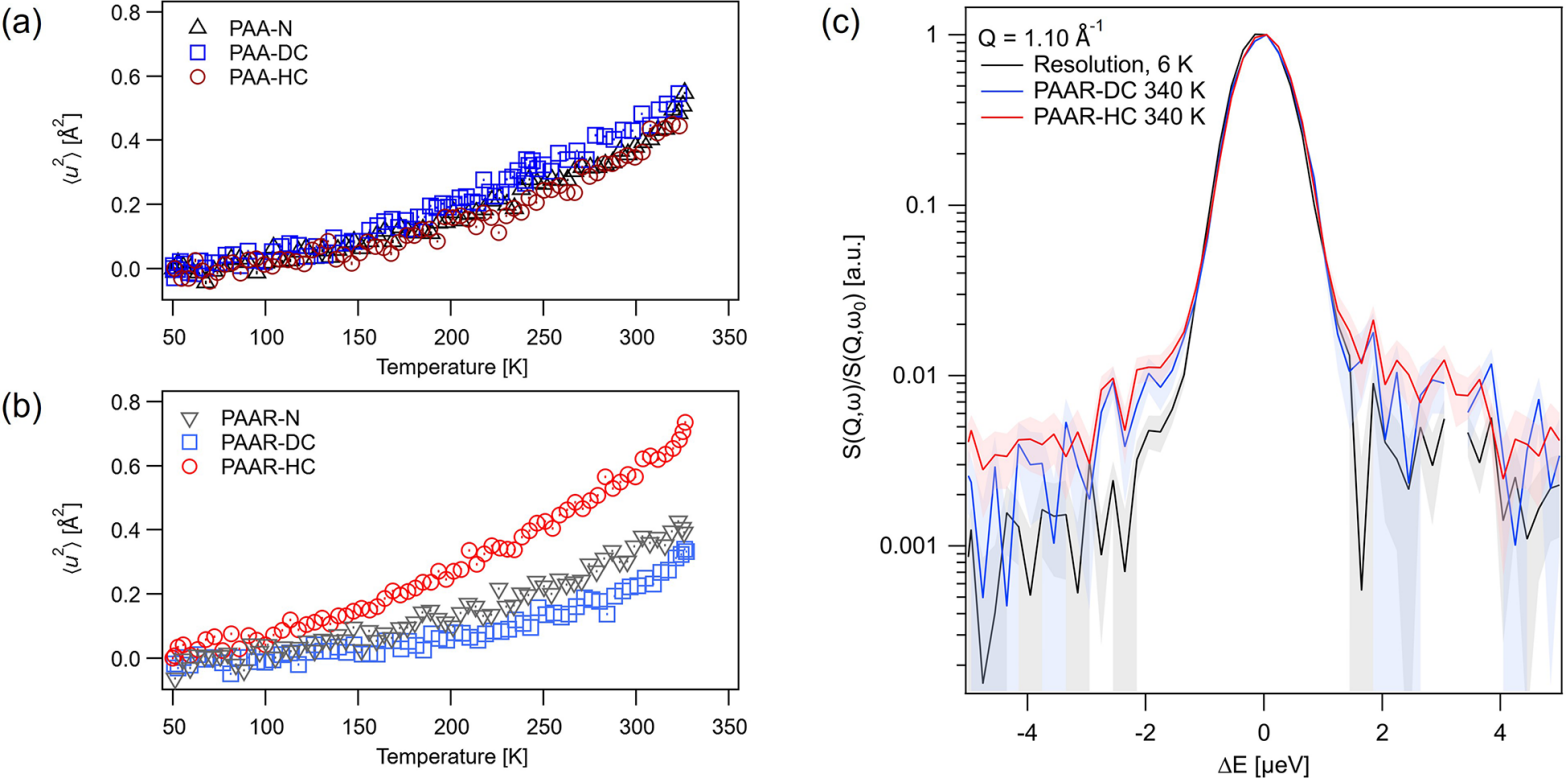
Mean squared displacement of the (a) PAA and (b) PAAR sample series as a function of temperature between 50 and 325 K. There is little change among the different dosing conditions in the PAA series, but more of an effect in the PAAR series. (c) Comparison of PAAR-HC and PAAR-DC sample scattering at 340 K to the resolution scattering (collected at 6 K) at *Q* = 1.10 Å^–1^. The elastic peak heights have been normalized to show there is very little quasi-elastic broadening. In (a,b) the 1 standard deviation error to the ⟨*u* ^2^(*T*)⟩ fits are smaller than the data points; in (c) the shading represents 1 standard deviation.

Surprisingly there is not an appreciable effect of dosing on the PAA series samples with the N, DC, or HC conditions; all of the ⟨*u* ^2^(*T*)⟩ curves generally overlap with one another as a function of temperature (Figure 3a). The PAA-N and PAA-HC curves overlap perfectly. The PAA-DC curve is slightly higher, but the differences are not significant with respect to the error bars. This is unexpected based on our previous QENS study examining PEI where we did see robust shifts in the ⟨*u* ^2^(*T*)⟩ values with these different dosing conditions.^17^ The lack of change in the PAA series with dosing indicates that polymer–sorbate interactions are not causing significant changes to backbone dynamics, nor are sorption products significantly contributing to ⟨*u* ^2^(*T*)⟩ within this time scale. This is generally consistent with our observations that both the water swelling (Figure 2a) and the stiffness (Figure S3) of the PAA did not change with the different dosing conditions. Taken together with the dosing independence of ⟨*u* ^2^(*T*)⟩ in Figure 3a, this suggests there is probably a strong hydrogen bonding network between the primary amines in the dry PAA that is not appreciably disrupted by either water or CO_2_ interactions.

In contrast, the PAAR sample series (Figure 3b) exhibits noticeable ⟨*u* ^2^(*T*)⟩ differences depending on the dosing condition at these time scales, especially above 120 K. Compared with the dry PAAR-N, the PAAR-HC (dosed with H_2_O and CO_2_) shows an appreciable increases in the magnitude of the mean square displacement; water and CO_2_ clearly facilitate the dynamics. However, in PAAR-DC (dosed with D_2_O and CO_2_) the mean square displacements decrease relative to PAAR-N. This indicates that the dynamics of just the polymer chain decrease when it is dosed with CO_2_ and water; the dynamics of water and any capture products (carbamates, bicarbonates) should be masked. We acknowledge that formation of bicarbonate or carbamate ions in the PAAR-DC system would generate either amino cations or ammonium groups on the polymer chains, and ionic interactions are known to suppress dynamics; this seems reasonable. However, it is striking that this did not occur in the PAA-DC sample where we know the carbamate ions induce cross-links between chains. In fact, the dynamics of the PAA-DC may have increased slightly above the dry sample (close to the error range). This might suggest, overall, that the strong hydrogen bonding network in PAA is essentially replaced by the ionic cross-links where the effects of strong hydrogen bonds or carbamate cross-links in the glassy state are equivalent. While we cannot ascertain this completely, it is an interesting observation.

In Figure 3b an onset anharmonic motion (nonlinear upward inflection) occurs between 100 and 150 K in the PAAR series. This is roughly 100 K below the onset of water motion (the so-called glass transition of bound or nonfreezing water) that occurs in many hydrated soft-matter systems.^27–29^ Since the PAAR-DC presumably does not have labile ^1^H moieties at the amine, we attribute this change in dynamics to polymer motion. Likewise, the PAAR-N sample should also be dominated by dynamics of the polymer backbone. It is possible there might be a proton hopping mechanism between amine sites that would contribute to the inelastic scattering, but it is unlikely that such a mechanism is active at 100 K as the binding energies of bicarbonate or carbamate ions are estimated to be on the order of 50–80 kJ/mol.^30^ Likewise, calculations suggest that the energy barrier for primary amine rotation in PEI is about 18.4 kJ/mol, suggesting that these rotational motions are also not active in the PAA in this low-*T* regime.^31^ Therefore, we argue that the dynamics in the low-*T* regime Figure 3 primarily reflect low frequency (sub-ns) vibrations and relaxations along the backbone of the polymers. This changes at higher temperatures, above room temperature, where the diffusive motions of the water and the capture products are activated. This is clearly seen in Figure 3b where the dynamics of PAA-HC are substantial around 300 K.

With that said, it is interesting in Figure 3a that dosing does not affect the chain dynamics of the PAA series, even above 300 K, but it does in the PAAR series shown in Figure 3b. In our previous publication on PEI in the rubbery phase, we showed mechanical stiffening upon codosing of water and CO_2_ that we ascribed to carbamate–ammonium interchain interactions. In the PAA system, there does not appear to be significant change in the stiffness of the dosed sample relative to the undosed PAA-N sample, presumably because the polymer is already in a glassy state and ion-pairing interactions are not strong enough to disrupt the strong hydrogen-bonding networks between the primary amines. Formation of bicarbonate in the dosed PAAR series creates an untethered ionic group that can associate with both neighboring chains and the sorbed water in the system. We believe that the change in dynamics measured in the elastic intensity is a reflection of this polymer-sorbate ensemble, with ^1^H motions dominating the scattering. These observations are largely in-line with the proposed mechanisms of CO_2_ capture and transport proposed by Ho and co-workers,^14,15^ as drawn in Scheme 1, where PAA transports CO_2_ via hopping from the amine site to the site along the cross-linking carbamate moieties, while PAAR enables free bicarbonate diffusion that is not covalently linked to the polymer backbone.

In addition to the fixed window scans on Emu, we also performed full dynamic scans to quantify the time and length scales of the motions. A few examples of these quasielastic spectra for the PAAR-HC and the PAAR-DC samples at 340 K (well above the subambient regime) are shown in Figure 3c. Also shown is the spectrum of the same sample when it is cooled to 6 K, where we assume all motion to be frozen. The 6 K spectrum represents the energy resolution of the emu spectrometer. This clearly shows that there is a very small amount, but measurable, quasielastic broadening beyond the resolution function at 340 K. In Figure 3c, all of the spectra were normalized by the intensity of the elastic peak. In this representation, the PAAR-HC and PAAR-DC spectra in Figure 3c look the same. However, there are subtle differences in both the elastic peak intensities and the quasi-elastic broadening. Next, we will parametrize the quasielastic broadening at 340 K for the different dosing conditions to quantify the time and length scale of the motions.

Our quantitative analysis of relaxational dynamics is done by fitting *S*(*Q*,ω) profiles using the following model function

2 $$S\left(Q,\omega \right)=\left[A_{el}\delta \left(\omega \right)+A_{qe}L\left(\Gamma \left(Q\right),\omega \right)\right]⊗R\left(Q,\omega \right)+BG\left(Q\right),where\;L\left(\Gamma \left(Q\right),\omega \right)=\frac{1}{\pi }\left(\frac{\Gamma \left(Q\right)}{{\left(\hbar \omega \right)}^{2}+{\Gamma \left(Q\right)}^{2}}\right)$$

where *A*_el_ and *A*_qe_ are area coefficients to the delta function describing the elastic peak (δ­(ω)) and the Lorentzian function with half-width at half-maximum (Γ­(*Q*)) describing quasi-elastic scattering. The model also includes a flat background function, *BG*(*Q*). The scattering terms are convoluted with the resolution function of the instrument, *R*(*Q*,ω).

An example fitted Emu spectrum is shown in Figure 4a for the PAAR sample under different dosing conditions with the component fit functions (dotted and dashed lines) plotted along with the total fit. Given the dosing independence of the ⟨*u* ^2^(*T*)⟩ data in Figure 3a for the PAA series, we would not expect any different in the full inelastic spectra. The full-width half-max (fwhm) of the Lorentzian extracted from the fit is plotted as a function of *Q* in Figure 4b. The variations of the fwhm with *Q* in the PAAR-HC and PAAR-DC samples are almost identical, and to a first approximation, linear with *Q*. There does appear to be a slightly positive slope, which would indicate a diffusive process, but the magnitude of the slope is small. A slope of zero (no *Q*-dependence) would indicate a spatially confined or constrained motion; from this observation we simply state that the motions might be considered slightly diffusive at the nanosecond time scale. Furthermore, there are not significant differences between the PAA and PAAR samples in the energy of the motion (within one standard deviation) of the fit result. We calculate the elastic incoherent structure factor (EISF) from the fit parameters as the ratio of amplitude of the elastic scattering (*A*_el_) to the total (sum of elastic and quasielastic, *A*_qe_) scattering (eq 3) and plot the results in Figure 4c.

3 $$EISF=\frac{A_{el}}{A_{el}+A_{qe}}$$

**Figure 4:**
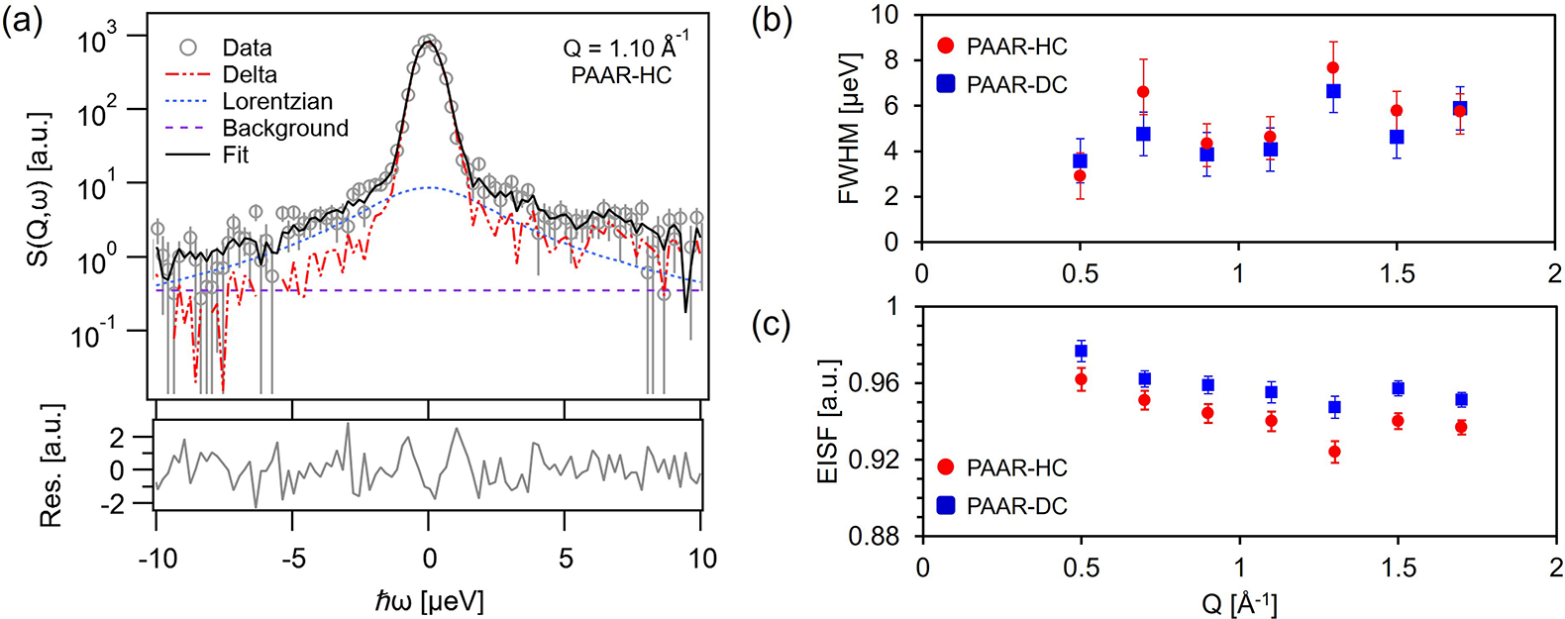
(a) Example scattering profile (open circles), fit components (dotted lines), total fit (solid line), and fit residuals (inset) for the PAAR-HC sample at 340 K and *Q* = 1.10 Å^–1^. Extracted (b) fwhm and (c) EISF parameters from the fit at each *Q* value for PAAR-HC (red circles) and PAAR-DC (blue squares) samples at 340 K. Error bars represent one standard deviation.

According to this equation, an EISF of 1 would indicate that the scattering is completely elastic (as would be the case at 6 K). In Figure 4c, we see that the EISF values for PAAR-HC and PAAR-DC are quite high, ranging from 0.93 to 0.98. It makes sense that the PAAR-HC is less elastic, given that the contributions from the sorbed water are also visible; PAAR-DC masks these contributions. These observations are generally consistent with the weak quasielastic scattering or strong elastic scattering seen in Figures 3c and 4a. This simply reflects the fact that there are not many dynamics in the window of the Emu spectrometer. For this reason, we turn to the larger energy range (faster dynamics) of the Pelican spectrometer.

The dynamics measured on Emu are roughly within the tens-of-ps to 1 ns time scale. A time-of-flight instrument, Pelican (also at ANSTO), provides a means to study faster dynamics in the approximate range of 1 to 63 ps. QENS profiles suitable for fitting were obtained at momentum transfers of *Q* = 0.45 Å^–1^ to 1.65 Å^–1^ binned in 0.2 Å^–1^ increments at temperatures of 300, 325, and 340 K for all samples. The data were fit from −2 to 1.5 meV. Similar to the procedure for Emu, we fit the data using a combination of functions including a delta function, δ­(ω), to capture the elastic scattering and a Lorentzian function capturing the quasielastic scattering, *L*_1_(Γ_1_(*Q*),ω). Attempts to incorporate a flat background, as was done with the Emu spectra, proved problematic. The quality of the fit could still be improved by adding a broad second Lorentzian. However, the fit parameters of the second Lorentzian and the amplitude of the flat background were highly correlated. As an alternative, we omitted the flat background and just used a broad second Lorentzian, *L*_2_(Γ_2_(*Q*),ω), with a minimum half-width half-maximum (HWHM) that is not allowed to drop below 1 meV to capture the background. This gave excellent fits with uniformly low residuals across the entire fitting regime (−2 to 1.5 meV) as shown in Figure 5a. The resulting *L*_1_ component fit with consistent trends in their HWHM values in the range of 0.1–0.3 meV, reflecting the lower frequency motions that may be associated with molecular transport (vide infra). We do not attempt to analyze trends in the broader *L*_2_ components that reflect very fast (sub-ps) local motions that we assume do not correlate with transport based on the time scale of diffusive dynamics. The prefactors *A*_el_, *A*_qe1_, and *A*_qe2_ describe the elastic and quasielastic scattering functions as denoted by subscripts -el and -qe, respectively. The model is described by the following equation

4 $$S\left(Q,\omega \right)=\left[A_{el}\delta \left(\omega \right)+A_{qe1}L_{1}\left(\Gamma_{1}\left(Q\right),\omega \right)+A_{qe2}L_{2}\left(\Gamma_{2}\left(Q\right),\omega \right)\right]⊗R\left(Q,\omega \right),where\;L\left(\Gamma \left(Q\right),\omega \right)=\frac{1}{\pi }\left(\frac{\Gamma \left(Q\right)}{{\left(\hbar \omega \right)}^{2}+{\Gamma \left(Q\right)}^{2}}\right)$$

**Figure 5:**
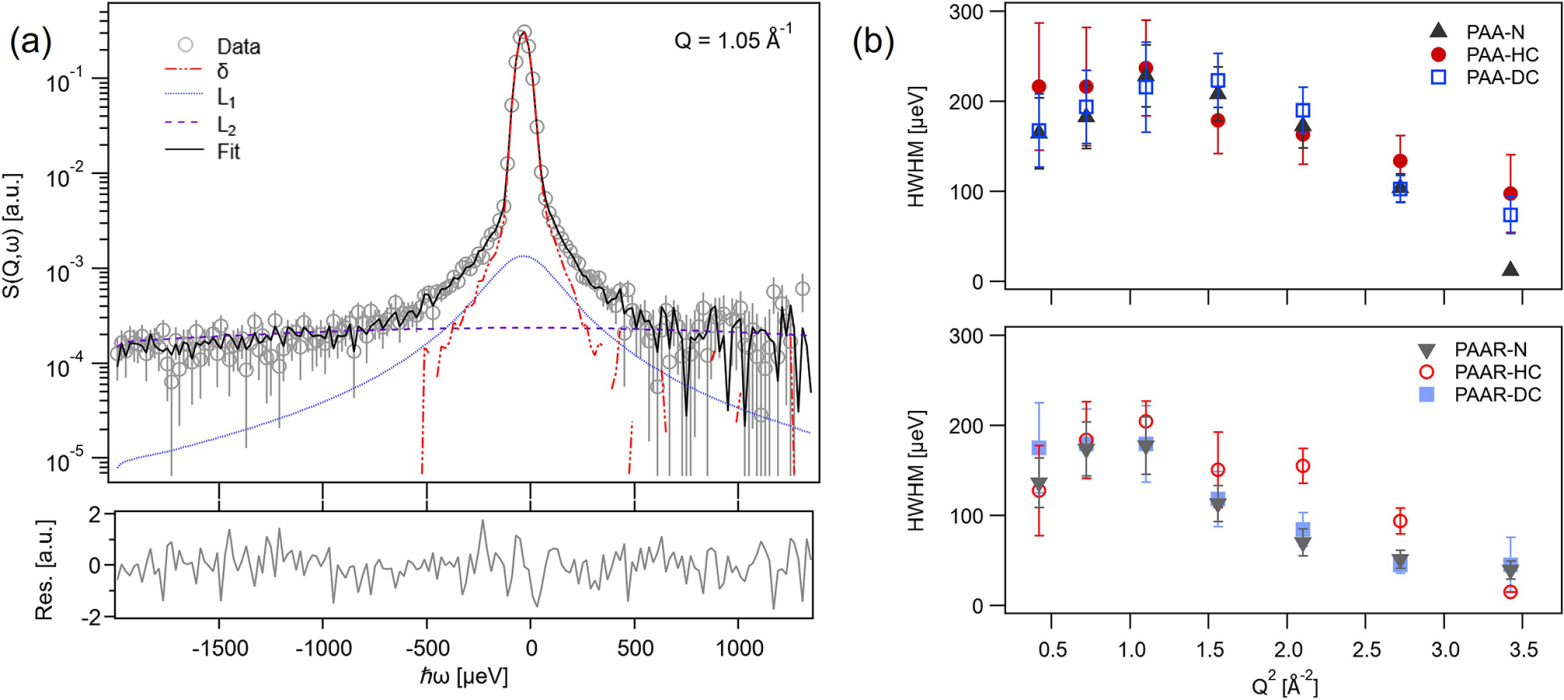
(a) A representative QENS spectrum and composite fit functions. (b) Plots of the *L*_1_ HWHM values obtained from fitting spectra as a function of *Q* ^2^. Data obtained at 340 K, error bars represent one standard deviation.

A representative spectrum and fit using this model are shown in Figure 5a. The half-width half-maximum of the narrower of the two Lorentzian functions (defined as *L*_1_) shows a nonlinear dependence with *Q* ^2^ that is indicative of a jump diffusion process as shown in Figure 5b. We omit the first fit data point at *Q* = 0.45 Å^–1^ due to poor fit confidence; however, it is included in Figure S4. The shape of the profiles in Figure 5b increases toward a peak around 1 Å^–2^ and then decays with increasing *Q* ^2^, rather than plateaus from the peak value. The decay in the profile at high *Q* ^2^ suggests the use of the well-known Chudley–Elliot (CE) jump diffusion model, where molecules jump between neighboring sites separated by a well-defined fixed distance after residing for a characteristic amount of time in each site.^32–34^ More importantly, the CE model contrasts strongly with the simple Fickian diffusion model where the molecules show a continuous random-walk motion which results in linear relationship between HWHM and *Q* ^2^. In our previous QENS work on PEI under these same dosing conditions, the Fickian diffusion mechanism was operative. However, those materials were measured above their glass transition, which naturally leads to Fickian diffusion. For glassy materials, like the PAA and PAAR materials presented here, the CE model with a well-defined jump length seems to adequately parametrize the data. The HWHM values of *L*_1_ as a function of *Q* are fit to the CE model based on the assumptions that the groups of atoms collectively vibrate in a given site for a duration time, τ, often called a residence time, before making a jump to another site situated a distance *l* away. We fully admit that there may be other diffusive models that are more appropriate, but the CE model gives us a reasonable platform to compare the different materials. In the CE model, if the jumps can occur in any direction, such as in a liquid or amorphous system, then the HWHM can be expressed by^35^

5 $$HWHM\left(Q\right)=\frac{\hbar }{\tau }\cdot \left(1-\frac{\sin\left(Ql\right)}{Ql}\right)$$

The HWHM vs *Q* data are fit using eq 5 between *Q* = 0.65 Å^–1^ and *Q* = 1.45 Å^–1^ for all samples and temperatures. A representative fit of PAA-HC and PAAR-HC at 325 K is provided in Figure 6 using the model described by eq 5. The residence times extracted using this model are presented in Table 2. In general, fitting results for the unfunctionalized PAA samples conditioned to various atmospheres yield residence times between 12 and 15 ps. The residence times for the PAA-HC sample does not exhibit an appreciable trend with changing temperature within the uncertainty of one standard deviation, while the PAA-N and PAA-DC samples do follow a slight increase in residence time with increasing temperature. Normally, a thermally activated diffusion process would lead to shorter residence times with increasing temperature. However, here this intuition is complicated by the fact our reaction products are exothermic, and heating could reverse the reaction, leading to unequal chemical environments at each temperature. The HWHM in the -HC dosed samples could be comprised of contributions from H_2_O, the polymer itself, CO_2_ capture products, and intermolecularly associated combinations of these components, whereas dynamics in the -DC and -N series are more likely to be predominantly comprised of polymer motions. Longer residence times with increasing temperature in the -N and -DC samples might suggest that the capture products are reversing and leading to stronger associations of the unreacted components to the polymer backbone. Since the trend is observed for both dosed (-DC) and undosed (-N) samples, we attribute the stabilization to polymer–polymer interactions that increase with increasing temperature in the regime studied here. In the -HC results where the temperature dependence is more flat, the additive motions of sorbed species (that we would expect would show decreasing residence time with increasing temperature) may be counteracting the polymer contribution observed in –N and -DC samples.

**Figure 6:**
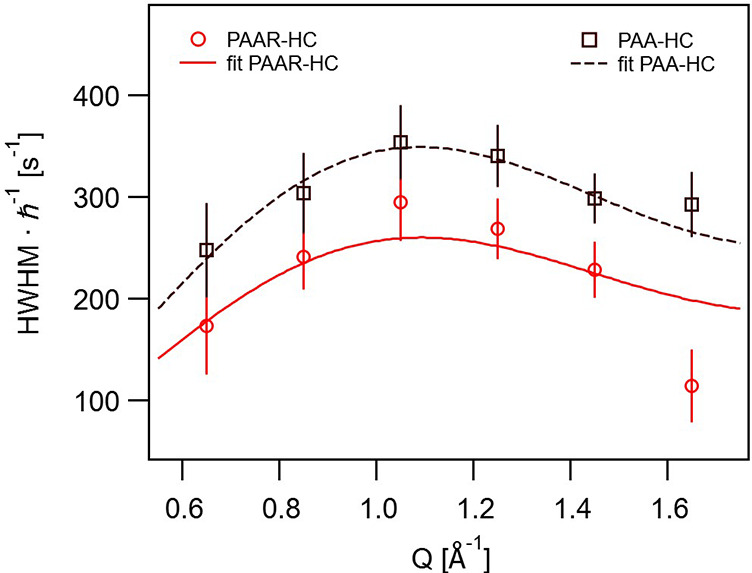
*L*_1_ HWHM data and Chudley–Elliot jump-diffusion model fits for PAA-HC (black squares and dashed line) and PAAR-HC (red circles and solid line) at 325 K. The fit model was applied between *Q* = 0.65 Å^–1^ and *Q* = 1.45 Å^–1^.

**Table 2:** Residence Time Extracted from the CE Model on Dosed Polymer Samples from QENS Spectra Measured on Pelican^a^

	residence time (τ, [ps])					
*T* [K]	PAA-HC	PAA-DC	PAA-N	PAAR-HC	PAAR-DC	PAAR-N
340	14.4 ± 0.7	14.5 ± 0.5	14.0 ± 0.5	17.1 ± 0.9	21 ± 2	22 ± 2
325	13.9 ± 0.7	13.4 ± 0.6	14.6 ± 0.7	18 ± 1	19 ± 1	17 ± 2
300	15.0 ± 0.9	11.4 ± 0.6	12.0 ± 0.8	15.6 ± 0.9	18 ± 2	12 ± 2

a Uncertainty represents one standard deviation.

It is notable that the residence times for the PAAR series are, in general, longer by 4 ps on average than the PAA samples across all temperatures and dosing conditions. A longer residence time suggests a higher energetic barrier to motion in PAAR versus PAA samples, which, if motion is attributed to the polymer, is consistent with the addition of the bulky isopropyl group to the system. Mirroring the observations from the PAA series, the residence times extracted for the PAAR-HC sample are nearly flat with increasing temperature (within calculated uncertainty), while the PAAR-N and PAAR-DC samples show an upward trend with increasing temperature.

The jump lengths extracted from the fits are listed in Table 3. The distance, *l*, represents the distance between jump sites where molecules vibrate locally for the duration of the residence time. The jump length in crystalline materials is well-defined as the atomic positions are well ordered. In amorphous systems, the jump lengths are not as discrete as there are more degrees of freedom that result in favorable conformations defining the landing “sites”. Considering the structure of neutral polyallylamine, the amine appendages can be between 3 Å and 6 Å apart from one another. The measured jump length values are within this distance (roughly 4 Å in PAA and between 4 Å and >5 Å in PAAR), so it is likely that the CE process represents fluctuations or reorganizations regulated by the amine groups. In PAA, this is plausible given that the carbamate mechanism binds two neighboring amines with a single CO_2_. It would be reasonable to see the polymer motions gated by the amine-to-amine distances, as the carbamate ions need to be in a paired state. The PAAR series has slightly larger and less precise jump lengths than the PAA series. The fact that the jump lengths tend to increase makes sense with the bicarbonate mechanism that creates free ions that are not coupled to the chain, thereby creating a broader distribution of interacting conformations. Nevertheless, the slightly larger jump lengths measured in PAAR contribute to an increased diffusion coefficient. Following a (*DQ* ^2^) relationship, the diffusion coefficient (*D*) can be estimated from the CE parameters using the relationship in eq 6 ^32,35^

6 $$D=\frac{l^{2}}{6\tau }$$

**Table 3:** Jump Length Extracted from the CE Model on Dosed Polymer Samples from QENS Spectra Measured on Pelican^a^

	jump length [*l*, Å]					
*T* [K]	PAA-HC	PAA-DC	PAA-N	PAAR-HC	PAAR-DC	PAAR-N
340	4.7 ± 0.3	4.2 ± 0.2	4.7 ± 0.2	5.5 ± 0.4	5.1 ± 0.6	5.0 ± 0.8
325	4.1 ± 0.3	4.1 ± 0.2	4.4 ± 0.2	4.6 ± 0.4	4.1 ± 0.3	3.9 ± 0.7
300	4.5 ± 0.4	3.9 ± 0.2	4.0 ± 0.4	4.0 ± 0.3	4.0 ± 0.6	5.0 ± 1.5

a Uncertainty represents one standard deviation.

The results are listed in Table 4 with error propagation from the *l* and τ parameter uncertainties obtained from fitting with eq 5. In our previous QENS studies on branched PEI at temperatures well above its glass transition, we found that water enhanced the center of mass diffusion and that the same motion was slowed upon addition of water and CO_2_ together (matching the -HC or -DC cases in this study).^17^ We do not observe such increased mobility imparted by water on the polymer in the PAA or PAAR samples as there is not much difference between the –N and -DC samples in either series based on our data. If there were appreciable plasticization or stiffening effects on the polymer itself, they would manifest as a change in dynamics between –N and -DC samples, as the polymer backbone is the main neutron scatterer (containing H) in these experiments. This observation echoes our dissipation results discussed earlier, where neither PAA nor PAAR samples show large mechanical stiffening/softening differences when comparing the film under N_2_ and the film dosed with humidified CO_2_. There is some evidence of an effect of dosing observed for the PAAR-HC sample, which shows water itself contributes to a shorter residence time in the -HC- vs -DC-dosed samples at all temperatures. The shorter residence times observed only in the PAAR-HC sample suggest that water protons are participating in the diffusion process being measured, such as in a proton transfer process.

**Table 4:** Diffusion Coefficients Calculated from the CE Model Parameters for Jump Length and Residence Time^a^

	*D* [×10^–5^ cm^2^/s]					
*T* [K]	PAA-HC	PAA-DC	PAA-N	PAAR-HC	PAAR-DC	PAAR-N
340	2.6 ± 0.3	2.0 ± 0.2	2.6 ± 0.3	3.0 ± 0.5	2.5 ± 0.5	1.9 ± 0.6
325	2.0 ± 0.3	2.0 ± 0.2	2.2 ± 0.3	1.9 ± 0.3	1.5 ± 0.3	1.4 ± 0.6
300	2.3 ± 0.4	2.3 ± 0.3	2.9 ± 0.5	1.7 ± 0.4	1.5 ± 0.4	3 ± 2

a Uncertainty represents one standard deviation.

While the CE diffusion mechanism appears to be the same in both PAA and PAAR, there are slight differences in the fitted parameters that could manifest in macroscopic effects. In summary, we find that (i) the jump lengths are longer and less discrete in PAAR compared with PAA and (ii) the residence times are longer in PAAR compared with PAA. Therefore, any increased diffusivity in PAAR samples must be attributed to the longer jump length and not the residence time (as a longer residence time would lower the diffusion coefficient).

From PM-IRRAS data, we know that the PAA sample contains amine-bound carbamate as the CO_2_ capture product. The carbamate moiety remains bound to an amine site and cannot freely diffuse through the polymer whereas PAAR forms both a carbamate and a bicarbonate product. The bicarbonate product is not tethered to the amine group, although it may have some attraction, and is more able to freely hop from site to site in the polymer matrix. While we do not believe we are capturing discrete diffusion of bicarbonate ions in these measurements, it is likely that cooperative motions between water, polymer, and ionic sorption production give rise to the apparent differences in dynamics. In practice, we believe this manifests as an increase in the CO_2_ permeance as Ho and Zhao reported values of 55 ± 2 Barrer in PAA and 297 ± 13 Barrer for PAAR (where 1 Barrer = 3.35 × 10^–16^ mol m m^–2^ s^–1^ Pa^–1^) measured for composite membranes tested for flue gas separation (110 °C with 20% CO_2_, 40% H_2_, and 40% N_2_ feed gas held at 58% RH).^14^

As mentioned previously, if we assume that the dynamics of the PAA-DC and the PAAR-DC reflect of the hydrated polymer backbones, the difference between these spectra and the PAA-HC and PAAR-HC analogous should reflect dynamics of the mobile water and capture products. To quantify this, we treated the fits for the -DC files as “resolution function” and then fit the resolution to the respective -HC files. Using this approach, any additional scattering above the resolution function on the -HC files should reflect the dynamics of the water and capture products. These fits are shown in Figure S5 of the Supporting Information for the PAA and PAAR samples at 340 K, where the quasielastic scattering is the strongest. An inspection of the residuals of the fits seems to support the addition of a narrow process centered around the elastic line. The addition of a Lorentzian does improve the quality of the fits. However, the intensity of this Lorentzian is within the noise of the residual. The data do not statistically support this additional level of interpretation even though it is clear that a weak process is present. It is likely that if the background motions of the hydrated polymers could be reduced by deuterium substitution of the PAA and PAAR, this process could be resolved.

## Conclusion

In this study, we combine measurements detailing the sorption behavior and dynamics of two polyallylamine CO_2_-transport membranes. The chemical uptakes of CO_2_ and water are qualitatively monitored by PM-IRRAS, elucidating the sorption products as carbamate or bicarbonate depending on the functionalization of the amine. The PAA sample containing only primary amines exclusively forms carbamate in the presence of humid CO_2_, while the isopropyl-functionalized amines in PAAR can form both bicarbonate and carbamate products. The separation of these products is distinct from the infrared spectroscopy technique. Using a tandem QCM/PM-IRRAS, we can calculate the specific water and CO_2_ uptake masses of both PAA and PAAR samples. The results of these experiments demonstrate a likely competition for sorption sites between H_2_O and CO_2_ in these polymer systems as well as insight that water within the polymer, rather than water cosorbing in the film, is consumed during bicarbonate formation in PAAR.

Beyond sorption experiments, we also examine the effects of dosing atmosphere on dynamics using elastic and quasielastic neutron scattering. In elastic scattering experiments conducted on the Emu backscattering spectrometer, we find that there is little influence of dosing on PAA in the subpicosecond to few-nanoseconds dynamic window. PAAR, in contrast, does show an increase in dynamics when exposed to H_2_O and CO_2_ (presumably from mobile protonated species, including water), and a slight decrease in dynamics associated with the polymer–sorbate system, evidenced by the sample dosed with D_2_O and CO_2_. Dynamic differences are not apparent in the quasielastic scattering of the same samples on Emu. The dynamic motions are additionally examined in a faster dynamic range on the Pelican time-of-flight instrument (1–63 ps) where a Chudley–Elliot model of jump diffusion well-defines one of the fit components. From the model, we extract residence times and jump lengths that differ between the PAA and PAAR series. The PAAR samples generally exhibit longer residence times between jumps and appear to have slightly larger jump lengths. While the sensitivity to ^1^H in these neutron experiments renders elucidation of the bicarbonate mobile carrier’s specific dynamics infeasible, the effects of dosing on complexation in a polymer-sorbate system are apparent.

## Supplementary Material



## Notes

Additional experimental details, data from TGA, QCM/PM-IRRAS, and dissipation measurements, and additional QENS fitting results (PDF)
