# Supplementary material for: Effect of Primary vs Secondary Amines on the Reactivity and Dynamics of CO2 in Polyallylamine Sorbents under Humid Conditions
Source: ACS Appl Polym Mater. 2026 Jun 22;8(13):10162–74. doi: 10.1021/acsapm.6c00437 (PMC13366487; doi:10.1021/acsapm.6c00437)
Supplement: Supplementary file 1 [file ap6c00437_si_001.pdf]

Supporting Information for

The Effect of Primary vs Secondary Amines on the Reactivity and Dynamics of CO_2 in Polyallylamine Sorbents Under Humid Conditions

Avery E. Baumann[1], Alice Klapproth[2], Richard A. Mole[2], Craig M. Brown[3], Christopher M. Stafford[1], Christopher L. Soles[1]*

[1]Materials Science and Engineering Division, National Institute of Standards and Technology, Gaithersburg, MD 20899, USA

[2]Australian Nuclear Science and Technology Organisation, New Illawarra Road, Lucas Heights 2234, NSW, Australia.

[3] Center for Neutron Research, National Institute of Standards and Technology, Gaithersburg, MD 20899, USA.

* csoles@nist.gov; christopher.soles@nist.gov

Experimental Methods

TGA

Thermogravimetric analyses (TGA) were conducted using a TA Instruments Q500 TGA. Roughly 10 mg of sample was conditioned at 80 °C prior to the TGA run. Mass loss was measured using the Hi-Res ramp mode (where the heating rate is slowed with increasing mass loss and raised with decreasing mass loss) with max rate of 50 °C/min from 80 °C to 600 °C in 100 mL/min flowing N_2, followed by a temperature ramp of 25 °C/min from 600 °C to 1000 °C in air.

SI Figures

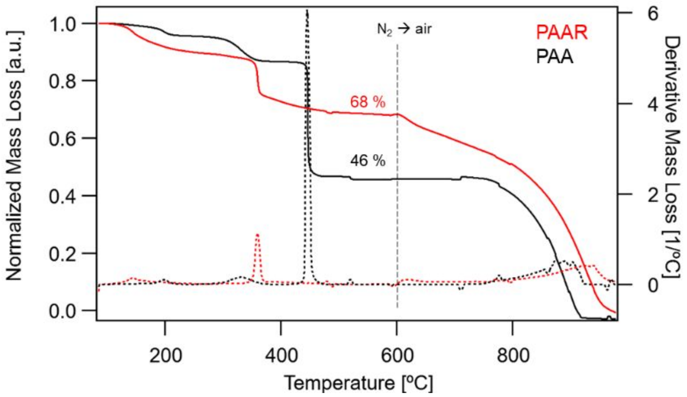

Figure S1. TGA curves for polyallylamine (PAA, black) and poly(N-isopropyl allylamine (PAAR, red) are shown on the left axis. The right axis corresponds to the derivative mass loss with respect to temperature and belongs to the dotted traces. The furnace atmosphere was switched from nitrogen to air at 600 °C, causing carbon residue oxidation. There is almost no inorganic residue after heating to 1000 °C in air.

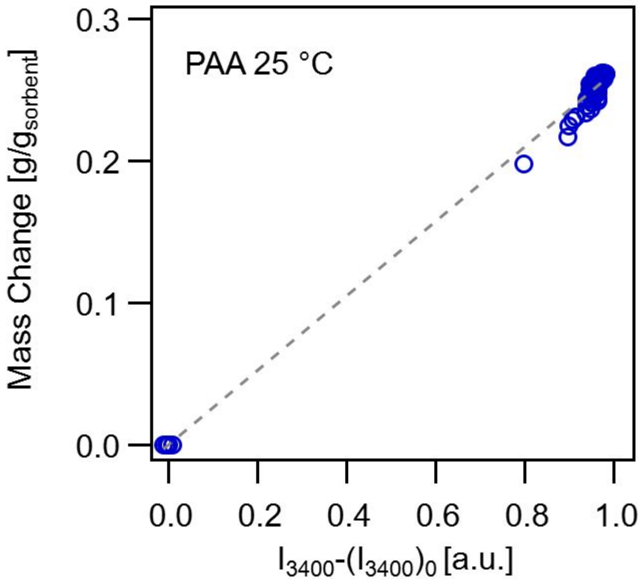

Figure S2. Change in mass versus change in polarization modulation infrared reflection absorption spectroscopy (PM-IRRAS) intensity at 3400 cm^{-1} (attributed to water O-H stretch) obtained during the tandem quartz crystal microbalance (QCM) and PM-IRRAS experiment under flowing humidified N_2. The initial PM-IRRAS intensity before introducing humidified N_2 was subtracted from all data to allow an intercept of 0. The linear fit of the data yields a slope of 0.263 ± 0.001 with a fixed y-intercept at 0.

The component mass of water sorbed in the humidified CO_2 experiment is calculated using the equation:

$$\left(M_{pH2O}\right)_{CO2+H2O} = \left[\left(\frac{M}{I_{3400}-(I_{3400})_0}\right)_{H2O} * \left(I_{3400} - (I_{3400})_0\right)_{CO2+H2O}\right] \quad \text{(eq. S1)}$$

where $\left(\frac{M}{I_{3400}-(I_{3400})_0}\right)_{H2O}$ is the slope of the trendline shown in **S3**. The PM-IRRAS intensity of the 3400 cm^{-1} peak in the mixed CO_2+H_2O experiments is multiplied by this slope-derived term to estimate the mass of water $\left(M_{pH2O}\right)_{CO2+H2O}$ in the sample. If other O-H or N-H stretches overlap with the 3400 cm^{-1} peak, the calculation would overestimate the component mass of water. The mass fraction of CO_2 is obtained from the difference of $\left(M_{pH2O}\right)_{CO2+H2O}$ and the total mass $(M)_{CO2+H2O}$:

$$\left(M_{pCO2}\right)_{CO2+H2O} = \left[(M)_{CO2+H2O} - \left(M_{pH2O}\right)_{CO2+H2O}\right] \quad \text{(eq. S2)}$$

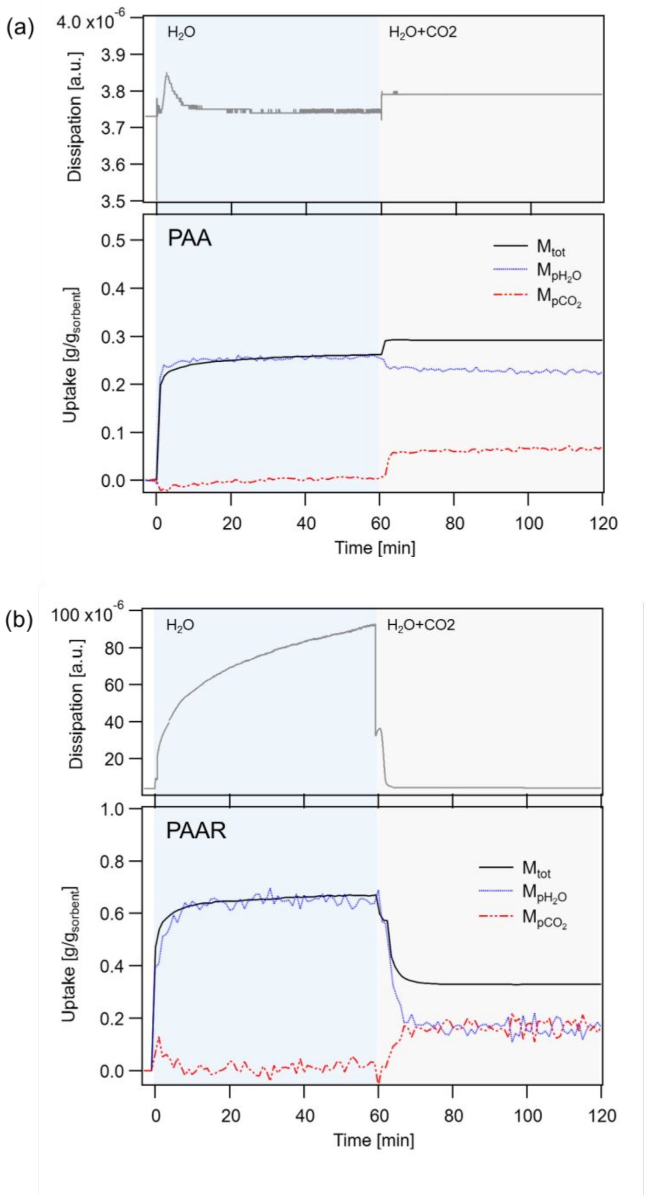

Figure S3. Dissipation data (top) alongside the mass uptake (bottom) measured via QCM for (a) PAA and (b) PAAR samples exposed to 1 h of humid N_2 and then 1 h of humid CO_2/N_2 mix (22 molar % CO_2 in N_2 balance, 57 % ± 5 % relative humidity achieved via H_2O bubbler).

In addition to the traditional mass uptake curves, the QCM instrument can also be operated in dissipation mode (D). In QCM-D, broadening of the HWHM of the sharp resonant peak frequency is used to quantify losses during the oscillations of the crystal. The complex electrical admittance of the resonator is broken down into its real, the conductance, and the imaginary, the susceptance, components to define the loss factor. More details of how this analysis is done can be found on the AWSensors tech note (https://awsensors.com/wp-content/uploads/2020/06/AWS-QCMD-Technology-2.pdf).

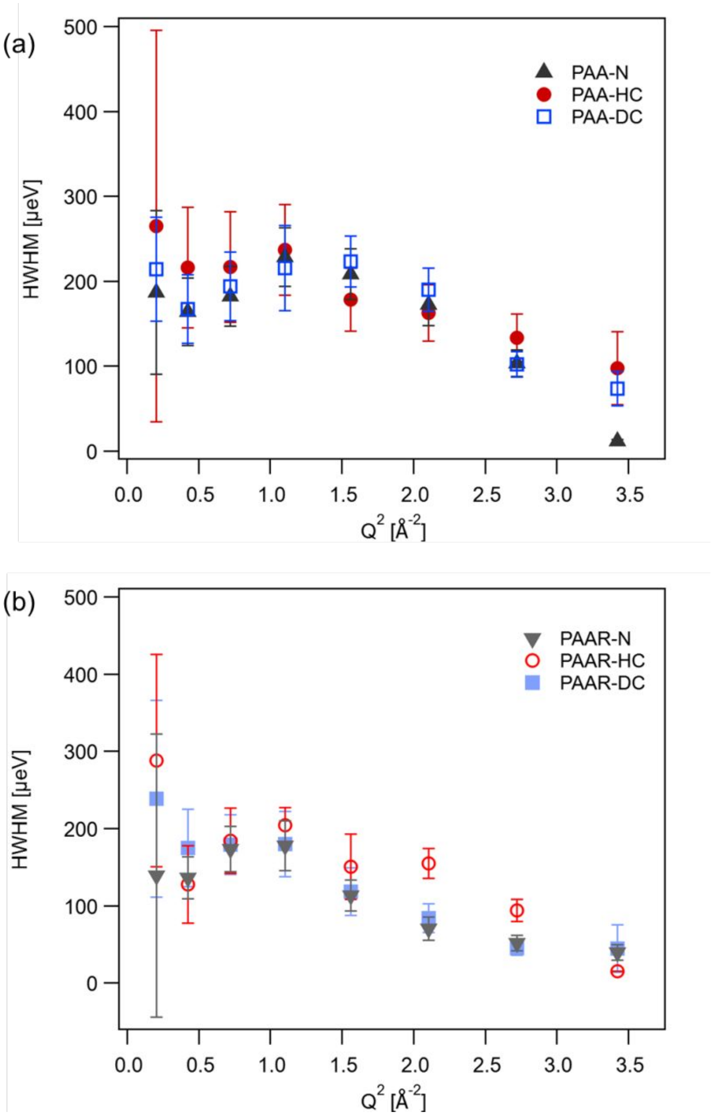

Figure S4. Full range of fitted L_1 half-width at half maximum (HWHM) vs. Q^2 data from Pelican experiments at 340 K.

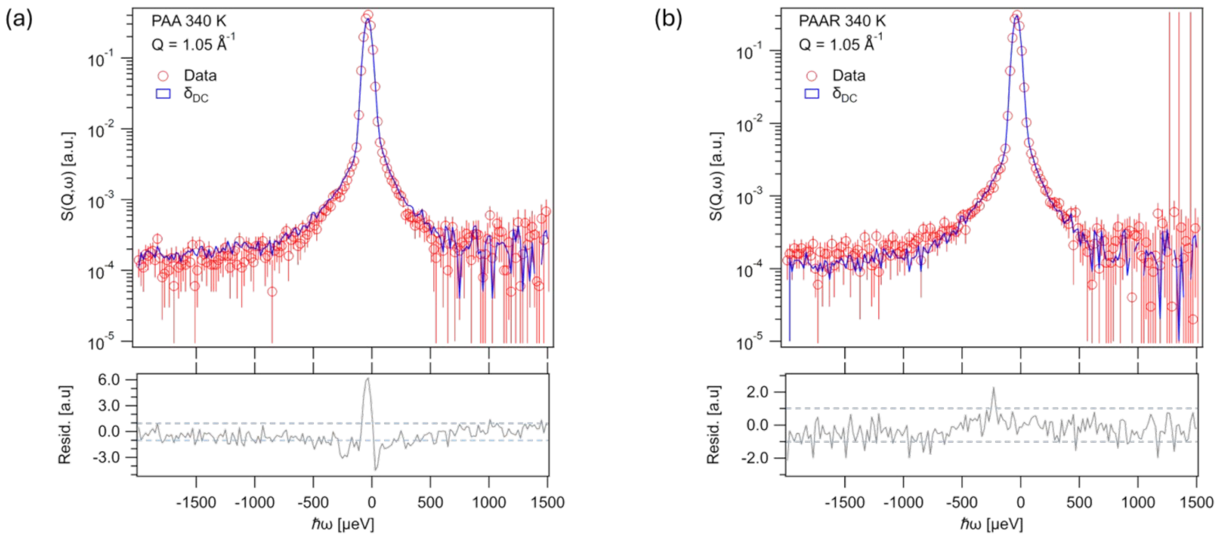

Figure S5. In this comparison, the experimental -HC spectra are fit using the fit from the -DC spectra as an apparent resolution function for (a) PAA and (b) PAAR samples at 340 K at Q = 1.05 $Å^{-1}$. The -HC data is fit by convoluting a single delta function (δ_{DC}) of variable height with the -DC fits to match the elastic peak intensities. In this representation, any additional quasielastic scattering can be assumed to stem from the mobile water and capture products. In each panel, the fit residuals could be improved by adding another Lorentzian centered around the elastic line, but the intensitiy of this Lorentzian would be comparable to the noise in the residual and not statistically supported.
